# Coldness or Darkness? Which Places Greater Stress on the Thyroid? Seasonal Changes in Thyroid-Stimulating Hormone and Thyroid Hormones

**DOI:** 10.3390/jcm13237293

**Published:** 2024-11-30

**Authors:** Abbas Ali Tam, Sevgul Fakı, Pervin Demir, Didem Ozdemir, Oya Topaloglu, Reyhan Ersoy, Bekir Cakir

**Affiliations:** 1 Department of Endocrinology and Metabolism, Faculty of Medicine, Ankara Yildirim Beyazit University, Ankara 06800, Turkey; sendidem2002@yahoo.com (D.O.); oyasude@gmail.com (O.T.); reyhanersoy@hotmail.com (R.E.); drcakir@gmail.com (B.C.); 2 Department of Endocrinology and Metabolism, Ankara City Hospital, Ankara 06800, Turkey; 3 Department of Biostatistics and Medical Informatics, Faculty of Medicine, Ankara Yildirim Beyazit University, Ankara 06800, Turkey; pervin.demr@gmail.com

**Keywords:** thyroid-stimulating hormone, free triiodothyronine, free thyroxine, season, temperature, light

## Abstract

**Background:** Although seasonal changes were suggested to be among the many factors that affect thyroid functions, this issue is still controversial. In this study, we aimed to investigate the possible relationship between seasonal changes and thyroid function. **Methods:** We retrospectively scanned all thyroid-stimulating hormone (TSH), free triiodothyronine (FT3), and free thyroxine (FT4) values checked in our hospital between 2019 and 2023. Using the big data approach, we examined the relationship between TSH and thyroid hormones and monthly and seasonal recorded climatic changes, particularly the duration of daylight and temperature. **Results:** A total of 195,534 serum samples were analyzed for TSH, 195,491 for FT3, and 195,487 for FT4. There were significant differences in the values of TSH, FT3, and FT4 between months (*p* = 0.001 for TSH, *p* < 0.001 for FT3 and FT4). The months with the highest levels of TSH, FT3, and FT4 values were January, December, and June, while the months with the lowest levels were July, May, and March-April, respectively. The differences between the maximum and minimum median values were 14.5% for TSH, 4.9% for FT3, and 5.7% for FT4. From January to August, as the temperature rose, there was a decrease in TSH values. Between September and December, as the temperature decreased, an increase in TSH was observed. **Conclusions:** This study revealed that TSH, FT3, and FT4 show seasonal variability and change in temperature is an important factor that plays role in this variability. It would be appropriate to take these changes into consideration when interpreting thyroid function tests.

## 1. Introduction

The rotation of the Earth around the sun causes seasonal changes, and adaptation to these changes is essential for living things to survive [1,2]. To cope with these seasonal fluctuations, animals change their behaviors, such as reproduction, migration, hibernation, immune function, metabolism, thermogenesis, growth, and molting [1,2,3]. Although temperature and precipitation may show annual changes, these provide unreliable seasonal information because there may be warm, rainy summers and relatively warm winters. Variations in day length are the most reliable seasonal signs since solstices and equinoxes occur around the same time every year. Thus, it is reasonable for organisms to use changes in photoperiod as a calendar [4]. In addition, biological clocks have emerged in many organisms for better adaptation to cyclic environmental changes. Almost all cells of the body have a circadian clock of approximately 24 h, and nearly all physiological functions and endocrine rhythms are regulated by this circadian system [5]. There is a complicated time regulation of the hypothalamopituitary (HPT) axis, which has pulsatile, ultradian, circadian, and circannual rhythms [6].

The effect of seasonal variations on the pituitary–thyroid axis in humans has been addressed in some previous studies. However, contradictory results were obtained and no clear conclusion could be reached. Despite studies showing that TSH increases in winter and decreases in summer, there are also studies reporting that it does not change. Similarly, both higher and lower FT3 was reported in summer in different studies. For FT4, there are publications stating that it is low in winter, low in summer, and also that it does not change with season [7,8,9,10,11,12,13,14]. The retrospective design of most studies, small sample sizes, and difference in the climate of the regions where the studies were carried out are the most frequently used explanations for the differences in the findings of the studies.

In this study, we aimed to investigate whether there are seasonal variations in TSH and thyroid hormones. We evaluated seasonally variable parameters such as temperature, duration of sunshine, duration of light, humidity, and atmospheric pressure during a four-year period using the big data approach.

## 2. Material and Methods

The medical records of patients of the Ankara Bilkent City Hospital Endocrinology and Metabolic Diseases outpatient clinic between February 2019 and May 2023 were examined retrospectively for the study. Patients over the age of 18 were included in the study regardless of whether they had thyroid disease or use levothyroxine or antithyroid drugs. The study was conducted with approximately 195,000 samples in 87,880 patients. The number of patients tested each month was consistent, with an average of 10,000 to 11,000 patients seen per month.

Blood samples for TSH, FT3, and FT4 were taken between 08:00 am and 16:00 pm. Ankara, where the study was conducted, is at an altitude of 891 m, at 39.9727′ latitude and 32.8637′ longitude, and is a city where the temperature drops below 0 °C degrees in winter and approaches 40 °C degrees in summer. For this province, the monthly average temperature (°C) (Mat), daily minimum temperature average (°C) (Dminta), minimum recorded temperature within the month (°C) (Minrtm), daily maximum temperature average (°C) (Dmaxta), maximum recorded temperature within the month (°C) (Maxrtm), monthly average air pressure (hPa) (Maap), monthly average relative humidity (%) (Marh), monthly average wind speed (m/s) (Maws), daily total sunshine duration average for the month (hours) (Dtsdam), minimum day duration (hours:minutes) (Mindd), and maximum day duration (hours:minutes) (Maxdd) were obtained from the Turkish State Meteorological Service. Then, the relationships between monthly and seasonal climate events and TSH, FT3, and FT4 were analyzed.

Serum TSH, FT3, and FT4 levels were determined using an immunoassay analyzer with the chemiluminescence method using acridinium ester technology (Atellica IM 1600 Analyzer, Siemens Healthcare Diagnostics, Tarrytown, NY, USA). The normal ranges for TSH, FT3, and FT4 were 0.55–4.78 mU/L, 2.3–4.2 ng/L, and 0.89–1.78 ng/dL, respectively. The limit of detection for TSH was 0.007 µIU/mL. It had a within-laboratory precision of ≤0.0032 mIU/L SD for samples of <0.020 mIU/L, ≤16% CV for samples from 0.020–0.299 mIU/L, ≤8% CV for samples from 0.300–90.000 mIU/L, and ≤10% CV for samples > 90.000 mIU/L. For FT4, the assay had a limit of detection ≤0.3 ng/dL. It had a within-laboratory precision of ≤0.03 SD for samples <0.5 ng/dL, ≤ 8.0% CV for samples from 0.5–1.0 ng/dL, and <6.0% CV for samples >1.0 ng/dL. The limit of detection for FT3 was ≤ 0.40 ng/L. The assay had a within-laboratory precision of ≤0.24 ng/dL SD for samples <2 ng/L, ≤12% CV for samples from 2.00–3.00 ng/L, ≤10% CV for samples from 3.10–4.40 ng/L, ≤15% CV for samples from 4.50–5.50 ng/L, and ≤15% CV for samples >5.50 ng/L. Euthyroidism was accepted as normal TSH, FT3, and FT4. Subclinical hypothyroidism and subclinical hyperthyroidism were defined as high and low TSH values, respectively, in the presence of normal FT3 and FT4. Overt hypothyroidism was defined as high TSH with low thyroid hormones, and overt hyperthyroidism was defined as low TSH with high thyroid hormones. The months of March, April, and May represented “Spring,” June, July, and August represented “Summer,” September, October, and November represented “Autumn,” and December, January, and February represented “Winter.”

## 3. Statistical Analysis

The distribution of variables was examined using Shapiro–Wilk’s test. The variables were summarized with mean ± standard deviation, median (25th–75th percentiles) or frequency (%). The values below/above the defined lower/upper detectible limits for variables were imputed using the “zCompositions” and “robCompositions” packages in R, respectively.

The values between months or seasons were compared by using the Kruskal–Wallis test, and a stepwise stepdown comparison was applied. A logarithmic transformation was applied to reduce the right skewness before analyzing covariance (ANCOVA) with adjustments for age and gender. The analysis of means comparing the mean-transformed rank of each group or the overall mean-transformed rank was applied to compare transformed values [Φ^−1^[0.5 + (ranked value/(2n + 1))]) in each month and season using the “ANOM” package in R. The value was significant when the transformed rank mean was outside of the upper definition line (UDL) and lower definition line (LDL).

To reduce the impact of multicollinearity on the regression model and to improve model prediction accuracy, Least Absolute Shrinkage and Selection Operator (LASSO) regression was used in the “caret” and “glmnet” packages in R software (Version 4.4.1). The results were obtained using a model based on the cosinor model, which considers seasonal variations through trigonometric functions. The other calculations were performed in SPSS version 21.0 (IBM Corp., Armonk, NY, USA). The statistical significance level was set at *p* < 0.05.

## 4. Examination of the Effects of Methodological Data on TSH, FT3, and FT4 Concentrations

The LASSO regression model results were evaluated to examine the effects of meteorological variables on TSH, FT3, and FT4 values while accounting for age, gender, and fixed effect of monthly variations (based on seasonal function), aiming to address the issue of multicollinearity. The explanatory coefficients for all three variables were found to be quite low in the models. The root mean square error (RMSE) values of TSH, FT3, and FT4 were 0.79, 0.10, and 0.11, respectively. Therefore, no predictions were made on the dependent variable because of the model, and the model remained undefined. However, the contribution of each independent variable, indicating the model contribution obtained from the glmnet model, could be assessed through the importance scores for each independent variable (Table 1). The coefficients indicated the impact of each variable on the response variable in the LASSO model. An increase in the variable had a positive (/negative) coefficient associated with an increase (/decrease) in the response variable. Table 1 provides a summary of the model, including relevant statistics (coefficients and ranking of the importance) for each predictor in the model. When age, gender, and cyclical effects were held constant separately for monthly and seasonal factors in the model, interpretations of the other methodological variable effects were made.

## 5. Results

The TSH values were obtained from a total of 87,880 patients, 73.5% of whom were female. The mean age was 49.57 ± 15.43 (median: 50, min–max: 18–99) years. A total of 195,534 serum samples were analyzed for TSH, 195,491 for FT3 and 195,487 for FT4. The medians for TSH, FT3, and FT4 were 1.59 mIU/L (Q1–Q3: 0.67–2.85), 3.21 ng/L (Q1–Q3: 2.90–3.55), and 1.18 ng/dL (Q1–Q3: 1.05–1.33), respectively.

### 5.1. Transformed Rank Mean Comparison in Each Month for TSH, FT3, and FT4 Levels

**Figure 1:**
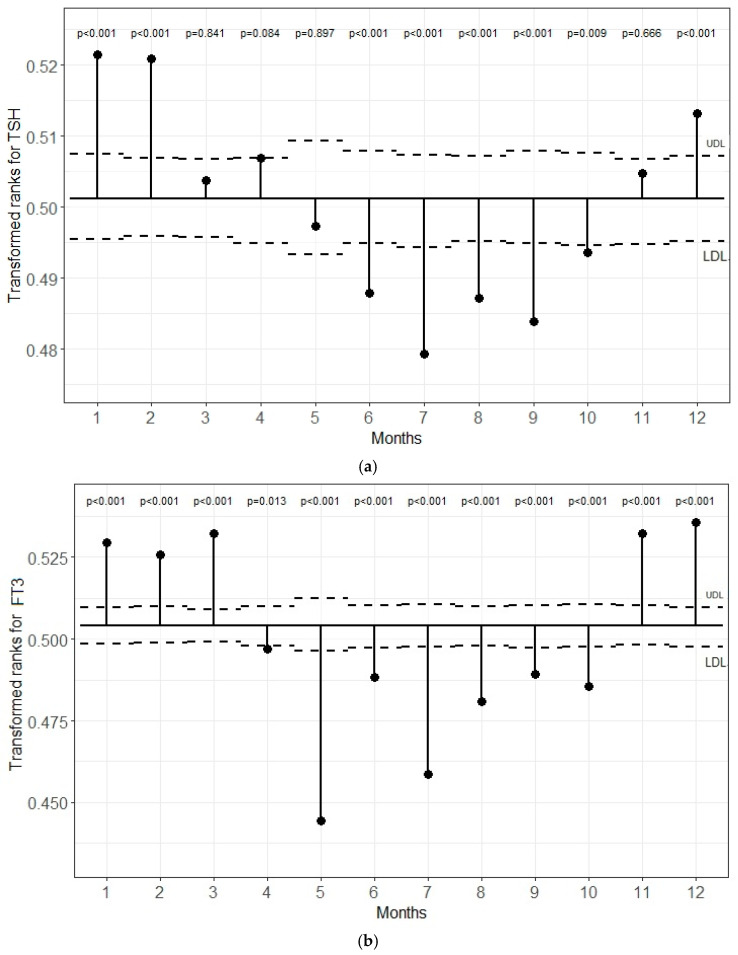

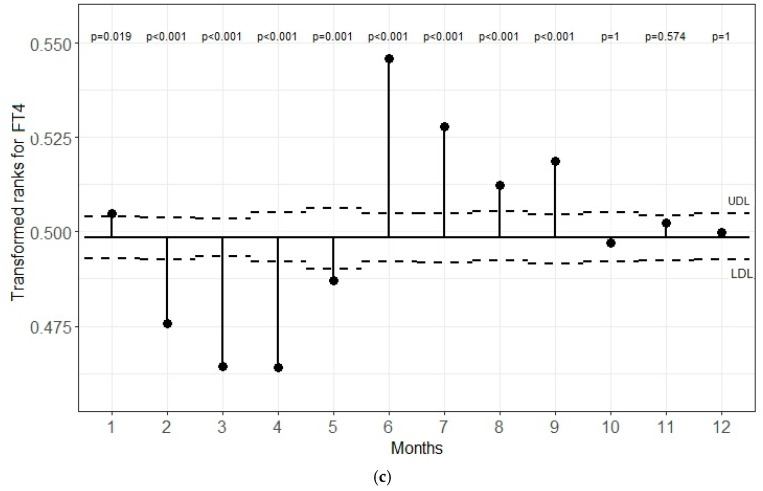
(**a**–**c**) Analysis of means (ANOM) with transformed ranks of monthly changes in TSH, FT3, and FT4 levels, respectively.

Figure 1a–c demonstrates the transformed rank mean in each month compared with the overall mean. There were significant differences in the months outside the lower and upper definition lines. The plot shows that the TSH levels were significantly lower in June, July, August, September, and October and significantly higher in December, January, and February than the overall transformed mean (*p* < 0.05). The FT3 values in November, December, January, February, and March were significantly higher than the overall transformed mean and, in other months, it was significantly lower (*p* < 0.05). When the FT4 values were compared for each month, the levels from February to May were lower and those in January and from June to September were higher than the overall mean (*p* < 0.05). The percent differences between the maximum and minimum median values were 14.5% for TSH, 4.9% for FT3, and 5.7% for FT4.

### 5.2. Seasonal Variations in Median TSH, T3, and T4 Levels

**Figure 2:**
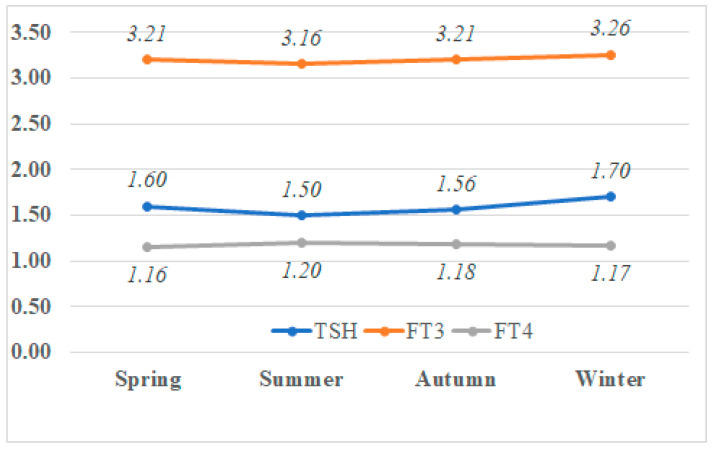
The seasonal median TSH (mIU/L), FT3 (ng/L), and FT4 (ng/dL) concentrations in each season.

The values of TSH, FT3, and FT4 were significantly different in at least one season from the other values (all *p*-values < 0.001) (Figure 2). For both TSH and FT4 values, differences were found in all comparisons. The seasonal ranking was as follows for TSH: winter > spring > autumn > summer. For FT4, it was summer > autumn > winter > spring. The FT3 value was similar for spring and winter, and the seasonal ranking was as follows: spring = winter > autumn > summer. Similarly, after conducting the ANCOVA analysis controlling for the effects of age and gender, there were significant differences in the median values of TSH, FT3, and FT4 between the seasons (*p* = 0.005, *p* = 0.008, and *p* = 0.003, respectively).

### 5.3. Transformed Rank Mean Comparison in Each Season for TSH, FT3, and FT4 Levels

**Figure 3:**
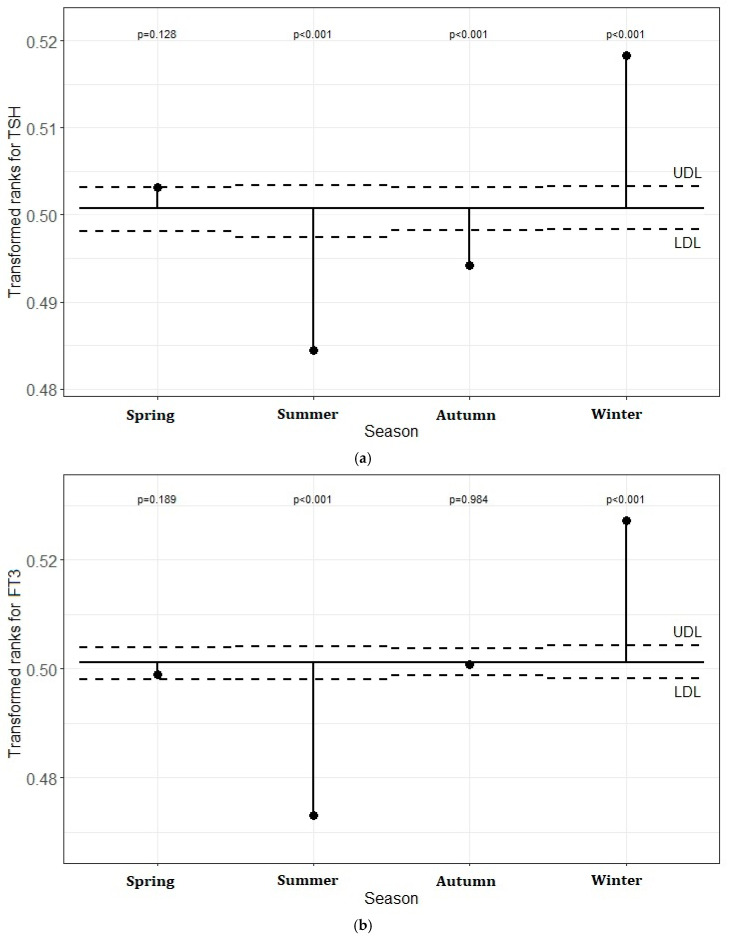

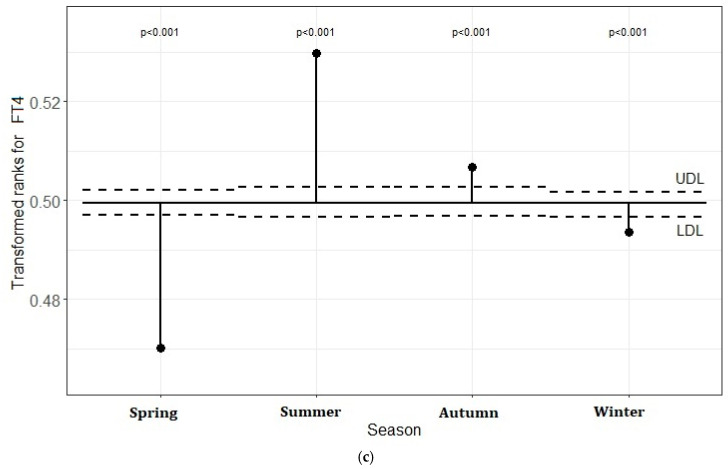
(**a**–**c**) Analysis of means (ANOM) with transformed ranks of seasonal changes in the TSH, FT3, and FT4 levels, respectively.

Figure 3a–c demonstrates the transformed rank mean in each season compared with the overall mean. There were significant differences in the seasons outside the lower and upper definition lines. The plot shows that TSH levels were significantly lower in summer and autumn and higher in winter than the overall transformed mean (*p* < 0.05). The FT3 values in summer were significantly lower than the overall transformed mean and they were significantly higher in winter (*p* < 0.05). When the FT4 values were compared in each season, the levels were higher in summer and autumn and lower in spring and winter than the overall mean (*p* < 0.05).

**Figure 4:**
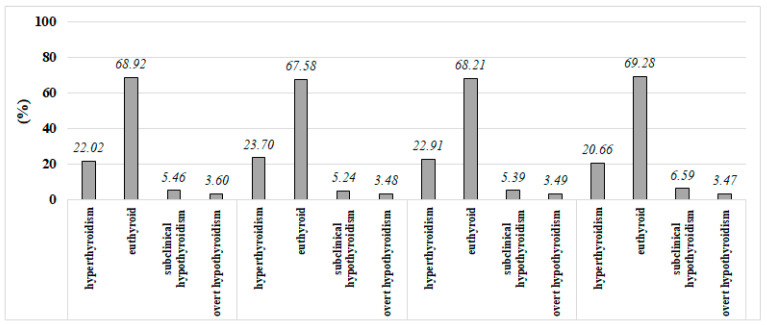
The distribution of disease groups in each season.

The distribution of functional disorders differed significantly by season (*p* < 0.001). The percentage of overt hypothyroidism in the seasons was similar (*p* > 0.05). The subclinical hypothyroidism percentage was higher in winter compared to other seasons (*p* < 0.05) (Figure 4).

#### 5.3.1. The Distribution of Monthly Average Air Temperature According to Season and the Values of TSH, FT3, and FT4

**Figure 5:**
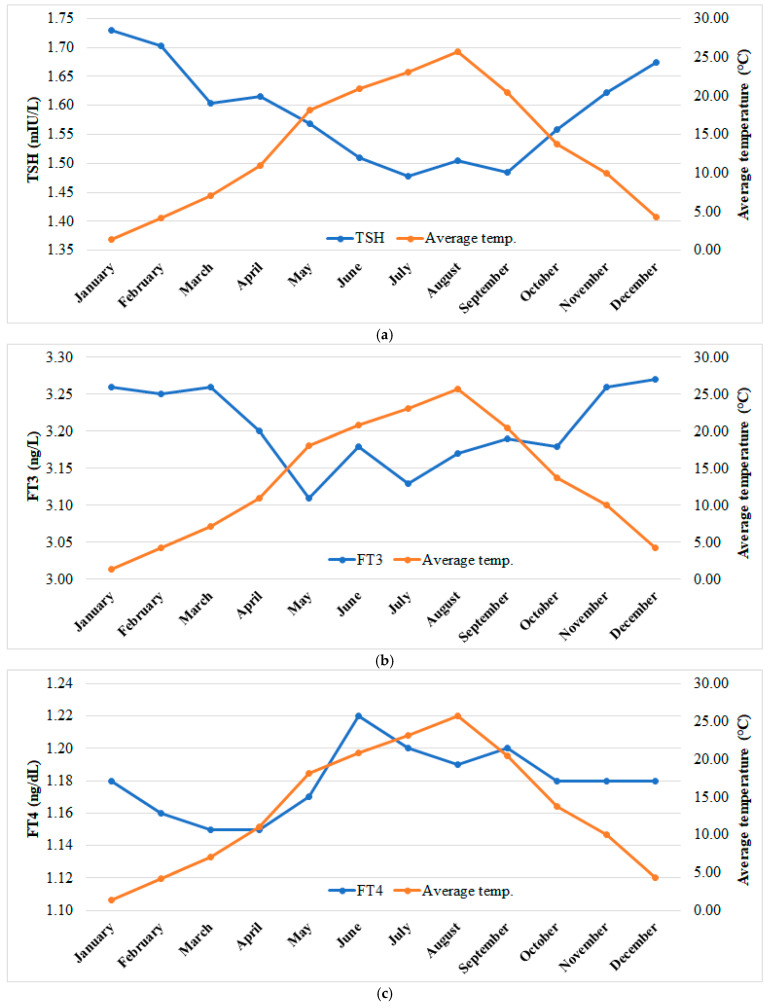
(**a–c**) The distribution of monthly average air temperature (°C) according to month and the median values of TSH (mIU/L), FT3 (ng/L), and FT4 (ng/dL).

An increase in air temperature was observed during spring and summer seasons, while a decrease was observed in the median values of TSH, FT3, and FT4. On the other hand, during autumn and winter seasons, as the temperature decreased, a slight increase was observed in TSH, while FT3 decreased (Figure 5a–c).

#### 5.3.2. Effects of Meteorological Data on TSH, FT3, and FT4 Concentrations

The impact of seasonal meteorological values on TSH: The coefficients were negative for five meteorological variables (Mat, Minrtm, Dmta, Maxrtm, and Marh) and positive for others (Dminta, Maap, Maws, and Dtsdam). The first three variables with the highest ranking of importance were identified as Dmta (93.15), Maxrtm (61.48), and Marh (47.06).

The impact of seasonal meteorological values on FT3 and FT4: When examining the impact of seasonal meteorological data on FT3, it was determined that the first four variables with the highest ranking of importance were Marh, Dtsdam, Dminta, and Minrtm. The variable with the most significant impact on FT4 was Dminta, with a weight of 100.0. Minrtm had a substantial importance, with a weight of 71.21. The third most important variable with a weight of 66.26 was Maxrtm (Table 1)

## 6. Discussion

Our study revealed that both TSH and FT3-FT4 show seasonal changes. TSH increased in winter as the temperature decreased and, in line with this, the frequency of subclinical hypothyroidism was increased in winter compared to other seasons. FT3 was higher in winter and FT4 was higher in summer compared to other seasons.

Among the studies using the big data approach, Yoshihara et al. [7]. examined seasonal changes in TSH and thyroid hormones over six consecutive years. They reported that TSH concentrations decreased during summer and increased during winter. In addition, TSH and FT3 were negatively correlated with daily temperatures. In a study including 1,506,495 subjects in Italy, it was shown that the mean TSH was higher in summer and winter seasons independently from age, gender, and environmental temperature. However, since there was no seasonal change for FT3 or FT4, the authors suggested that the seasonal change in TSH was independent from thyroid hormones, gender, age, and environmental temperature [10]. In the study conducted in Sulaymaniyah, Iraq, where there is a significant difference between summer and winter seasons and thus has a suitable environment to study hormonal variations, TSH and thyroid hormones were generally similar during summer and winter in euthyroid subjects and patients with subclinical hypothyroidism. However, there was a small but significant increase in FT3 in euthyroid subjects and a small decrease in FT4 in patients with subclinical hypothyroidism in winter. The duration of outdoor stay had an effect on only FT3 in euthyroid subjects during winter. While variations in climatic components had some effect on TSH and thyroid hormones in euthyroid subjects, they had no effect in patients with subclinical hypothyroidism [13].

Variation in environmental temperature is one of the most frequently suggested mechanisms to explain annual changes in TSH [12]. The mean temperatures, variations in temperatures throughout the year, sunshine hours, and humidity of countries are different from each other, as well as the prevalence of thyroid diseases [11]. In studies conducted with healthy individuals in China [15] and with those receiving levothyroxine treatment in Korea [16] and Japan [17], which have similar climates and where the temperature drops significantly in winter, a negative correlation was detected between TSH levels and environmental temperature. However, in two studies performed in Italy, which is warmer than Asia in winter, TSH levels and environmental temperature were not correlated [10,18]. This suggests that seasonal fluctuations might be more evident in cold climates [11]. Previous studies examining the effects of seasonal changes on humans were mostly conducted in polar regions where the temperature is freezing cold most of the time in winter and lower than the temperatures in temperate climates in summer. It was shown that serum TSH and FT3 increased and FT4 decreased in euthyroid subjects who lived for a long time in an extremely cold climate in Antarctica [7,13,15]. In another study, a 30–50% increase in TSH was observed in young subjects living for more than five months in Antarctica. Similarly, a 50% increase in serum TSH was also reported in patients with hypothyroidism and taking a fixed dose of levothyroxin during the winter months [19].

Although the underlying mechanisms of seasonal variations in TSH and thyroid hormones are not fully understood, different mechanisms have been suggested as to how season or temperature affects thyroid hormones. Firstly, it is known that the enzymatic activities of deiodinases are affected by temperature, which further affects thyroid hormone metabolism. A decrease in FT4 levels in cold temperatures may be related to increased type 2 deiodinase activity. However, it is expected that increased type 2 deiodinase activity would also increase FT3 levels [20]. Secondly, low temperatures stimulate the production of thyrotropin-releasing hormone (TRH) from the hypothalamus, which in turn leads to an increase in the production of TSH. At low temperatures, the rise in thyroid hormone levels accelerates metabolism, playing a crucial role in preserving the body’s core temperature [8]. The duration of exposure to temperature is also important. A study conducted on rabbits demonstrated that exposure to 4 °C for a duration of seven weeks enhanced the conversion of T4 to T3 [21]. O’Malley et al. demonstrated that a 30 min exposure to 4 °C induced a marked elevation in the concentrations of TSH, T3, and T4 in healthy individuals [22]. Lastly, temperature can affect the binding of thyroid hormones to plasma proteins and their clearance from plasma.

Adenohypophysis in humans consists of two main parts: the pars distalis (PD) and the pars tuberalis (PT). PD-derived TSH is regulated by a feedback system involving TRH and thyroid hormones. In contrast to thyrotrophs in the PD, those in the PT do not express receptors for TRH or thyroid hormones, rendering the regulation of PT-derived TSH independent of the hypothalamic–pituitary–thyroid axis. Instead, PT thyrotrophs are characterized by a high density of melatonin receptors, and the synthesis and secretion of PT-derived TSH are modulated by melatonin in mammals. Furthermore, while the bioactivities of TSHs derived from both the PT and PD are similar at the cellular level, PT-derived TSH interacts with immunoglobulins and albumin in the bloodstream to form macro-TSH complexes, leading to a loss of its bioactivity [3,5]. Although the exact prevalence of macro-TSH is not well established, it has been reported to be more prevalent in women and the elderly, with prevalence rates ranging from 0.6% to 3.1% [23].

Exposure to light is another factor that is thought to contribute to seasonal variations in thyroid function. Light is perceived by opsins, and the signal is transmitted to the suprachiasmatic nucleus (SCN), the central circadian pacemaker, via the retinohypothalamic tract. The SCN subsequently regulates the synthesis and secretion of melatonin by the pineal gland, which is released exclusively during the night [3]. Melatonin regulates various physiological processes through neuroendocrine pathways and plays an important role in the control of circadian or seasonal rhythm [7]. Melatonin also affects TSH secretion via M1 receptors in the pars tuberalis [5]. Animal studies have shown that it can negatively affect the secretions of the HPT–thyroid axis. Thus, through the suppressive effect on melatonin secretion, exposure to light may stimulate the axis [9].

In our study, the duration of light was among the most important parameters that affected seasonal changes in FT3 and FT4 in the regression analysis. There are studies with different results in the literature on this subject. Hassi et al. [24] stated that T3 changes with light and TSH changes with temperature. In addition, Mahwi et al. [13] found a positive correlation between the duration of sunshine and FT4. However, there were no associations between the duration of light and TSH, FT3, and FT4 in the study by Yoshihara et al. [7].

Thyroid dysfunctions are one of the most prevalent endocrine diseases and may cause serious consequences if not diagnosed and treated in time. Serum TSH is the most important parameter that is used to evaluate thyroid function and show the competence of the HPT–thyroid axis. Before deciding whether to treat a patient based on his/her TSH values, clinical factors such as gender, age, season, and ethnicity should be taken into account to determine the personalized reference range [15]. Ignoring possible fluctuations in TSH may lead to underdiagnosis or overdiagnosis of thyroid diseases and consequently under/overtreatment with thyroid medications [11]. Clinically, if normal ranges of TSH and thyroid hormones are not adjusted according to the season, small differences in findings may lead to misdiagnosis, which may result in unnecessary tests and treatments in some patients while not diagnosing the dysfunction in others [25].

In addition to the seasonal variations observed in thyroid function, numerous studies have highlighted the significant impact of seasonal changes on various endocrine and metabolic diseases, including cardiovascular disease, diabetes mellitus, and obesity. For instance, both type 1 and type 2 diabetes show poorer glycemic control during colder months [26], while cardiovascular events peak in winter due to factors like colder temperatures and increased blood pressure [27]. Similarly, weight gain is more common in winter, likely due to reduced physical activity and altered eating habits [28]. These effects should be considered when diagnosing and managing endocrine and metabolic disorders to avoid misdiagnosis and optimize treatment strategies.

Our study has some limitations. The most important ones are that it was retrospective and that data were collected from all patients, regardless of thyroid disease and medication use. In addition, the data were obtained from a single hospital. The lack of data on smoking, alcohol consumption, body mass index, diet, exercise, exposure to pollutants, vitamin D levels, and iodine status represents another limitation of the study. A significant portion of the study period coincided with the COVID-19 pandemic. During this time, strict measures, including lockdowns, were implemented. As a result, the majority of the population remained at home, likely staying awake until late hours, being exposed to artificial lighting, and experiencing changes in their eating habits. These factors may have influenced the measurements taken during this period. For instance, morning plasma TSH levels could be approximately twice as high in individuals who experienced sleepless nights compared to those who had a normal night’s sleep [5]. Furthermore, the effects of photoperiod and ambient temperature can be mitigated by the use of artificial lighting, heating, and air conditioning. Blood samples were routinely collected indoors and maintained at a constant room temperature. It is not easy to evaluate the real exposure of humans to climatic conditions. Studies conducted with animals show that the effects of climatic factors are significant. However, the technological opportunities we have in the modern world might moderate the impact of climate change on humans. Animals do not wear clothes or use air conditioners. On the contrary, a patient who comes to hospital in the winter may have spent the night at 25 degrees thanks to their heating system even though it is −5 degrees outside. It is highly probable that he/she will be under the influence of heating systems also on the way to and at the hospital. The opposite is also true in summer. As another limitation, macro TSH is not measured in our center, and we are unaware of the prevalence of macro TSH in our patients. On the other hand, our study has outstanding strengths. First, the data used in this study were derived from a continuous four-year period and included >195,000 samples. Also, the number of patients examined each month remained stable throughout the study period, with an average of 10,000 to 11,000 patients. This consistent sample size helps to reinforce the reliability and generalizability of our findings. Secondly, Ankara, where the study was conducted, is a city where four seasons are experienced and temperature differences are evident in summer and winter. In addition, we used the big data approach, which allowed us to minimize the impact of confounding factors such as sex, age, individual biologic variations, and the presence of thyroid dysfunctions [7].

In conclusion, our study reveals that there are seasonal changes in TSH, FT3, and FT4. In clinical practice, considering fluctuations in TSH and possible underlying mechanisms may provide a more accurate approach. Since climatic/seasonal changes are one of the factors that can also affect thyroid function, these should also be taken into consideration when making clinical decisions.

## Figures and Tables

**Table 1:** The ranking of the importance of each independent variable on the dependent variable.

	Variables	TSH		FT3		FT4	
		Coef	Importance	Coef	Importance	Coef	Importance
Monthly	Age	X		−0.024	100.00	0.001	
	Gender	−0.006	37.53	0.019	77.63	0.004	3.34
	Mat	X		0.00003	0.11	0.009	10.44
	Dminta	X		−0.002	6.58	0.008	9.67
	Minrtm	X		0.002	6.42	−0.002	1.75
	Dmta	−0.017	100.00	−0.006	26.31	0.001	0.29
	Maxrtm	−0.0002	1.03	0.010	41.00	−0.003	2.12
	Maap	−0.002	14.22	−0.004	16.01	−0.004	3.76
	Marh	X		0.023	94.77	−0.008	8.92
	Maws	−0.006	33.55	−0.001	4.06	0.005	4.57
	Dtsdam	X		0.016	66.92	−0.011	12.92
	Maxdd	X		0.012	49.76	0.003	2.34
	Mindd	X		−0.020	80.48	0.064	82.98
	Cosine component	X		X		0.077	100.0
	Sine component	0.005	31.73	0.0002	0.76	0.010	12.18
	Intercept	0.011		0.511		0.075	
Seasonal	Age	0.0004	0.12	−0.024	27.14	0.001	1.63
	Gender	−0.008	9.39	0.019	21.27	0.004	23.21
	Mat	−0.024	28.88	0.0004	0.00	−0.001	0.48
	Dminta	0.003	3.52	−0.030	33.39	−0.013	100.00
	Minrtm	−0.0003	0.00	−0.021	23.92	0.009	71.21
	Dmta	−0.075	93.15	−0.004	4.12	−0.001	0.00
	Maxrtm	−0.050	61.48	−0.011	11.79	−0.009	66.16
	Maap	0.014	16.44	0.020	21.90	−0.001	5.37
	Marh	−0.038	47.06	0.059	66.81	−0.007	49.75
	Maws	0.008	9.88	−0.005	5.03	0.007	48.00
	Dtsdam	0.005	5.71	0.040	45.63	0.004	24.73
	Cosine component	−0.081	100.00	−0.088	100.00	−0.002	7.83
	Sine component	−0.005	5.70	−0.004	4.69	−0.007	50.53
	Intercept	0.011		0.512		0.075	

Cosine and sine components represent the amplitude and phase of a cyclic pattern, respectively. The use of both sine and cosine terms is a common approach to model seasonality because it allows the model to consider both the amplitude (captured by the sine term) and the phase (captured by the cosine term) of the seasonal effect. The “X” in some variables indicates that the variable does not significant affect the dependent variable. Variables that have coefficients equal to zero, therefore, do not contribute to the model’s predictions. Mat: Monthly average temperature (°C), Dminta: Daily minimum temperature average (°C), Minrtm: Minimum recorded temperature within the month (°C), Dmta: Daily maximum temperature average (°C), Maxrtm: Maximum recorded temperature within the month (°C), Maap: Monthly average air pressure (hPa), Marh: Monthly average relative humidity (%), Maws: Monthly average wind speed (m/s), Dtsdam: Daily total sunshine duration average for the month (hours), Mindd: Minimum day duration (hours:minutes), Maxdd: Maximu day duration (hours:minutes).

## Data Availability

The datasets generated and analyzed in the present study are available upon reasonable request to the corresponding author.
